# A novel surface protein of *Trichomonas vaginalis *is regulated independently by low iron and contact with vaginal epithelial cells

**DOI:** 10.1186/1471-2180-6-6

**Published:** 2006-01-31

**Authors:** V Mundodi, AS Kucknoor, T-H Chang, JF Alderete

**Affiliations:** 1 Department of Microbiology, University of Texas Health Science Center, San Antonio, TX, USA

## Abstract

**Background:**

Trichomonosis caused by *Trichomonas vaginalis *is the number one, non-viral sexually transmitted disease (STD) that affects more than 250 million people worldwide. Immunoglobulin A (IgA) has been implicated in resistance to mucosal infections by pathogens. No reports are available of IgA-reactive proteins and the role, if any, of this class of antibody in the control of this STD. The availability of an IgA monoclonal antibody (mAb) immunoreactive to trichomonads by whole cell (WC)-ELISA prompted us to characterize the IgA-reactive protein of *T. vaginalis*.

**Results:**

An IgA mAb called 6B8 was isolated from a library of mAbs reactive to surface proteins of T. vaginalis. The 6B8 mAb recognized a 44-kDa protein (TV44) by immunoblot analysis, and a full-length cDNA clone encoded a protein of 438 amino acids. Southern analysis revealed the gene (tv44) of T. vaginalis to be single copy. The tv44 gene was down-regulated at both the transcriptional and translational levels in iron-depleted trichomonads as well as in parasites after contact with immortalized MS-74 vaginal epithelial cells (VECs). Immunofluorescence on non-permeabilized organisms confirmed surface localization of TV44, and the intensity of fluorescence was reduced after parasite adherence to VECs. Lastly, an identical protein and gene were present in Tritrichomonas foetus and Trichomonas tenax.

**Conclusion:**

This is the first report of a *T. vaginalis *gene (*tv44*) encoding a surface protein (TV44) reactive with an IgA mAb, and both gene and protein were conserved in human and bovine trichomonads. Further, TV44 is independently down-regulated in expression and surface placement by iron and contact with VECs. TV44 is another member of *T. vaginalis *genes that are regulated by at least two independent signaling mechanisms involving iron and contact with VECs.

## Background

The amitochondriate protist *Trichomonas vaginalis *is responsible for the number one incidence of sexually transmitted disease (STD) worldwide with ~9 million new cases of vaginitis in the US and 250 million cases worldwide [1-3]. There are serious health consequences for women, including adverse pregnancy outcomes, risk for cervical cancer, pelvic inflammatory disease, infertility, and infection by other STD agents [4-8]. Symptomatic women may experience foul-smelling discharge, severe irritation, and abdominal pain while some men will have a non-gonococcal, non-chlamydial urethritis. Trichomonosis is now appreciated to increase the portal of entry and exit for HIV [9,10], and the disproportionate incidence of this STD among American minorities is evidence that this STD is a health disparities disease. Equally troubling is that unlike other STDs like Chlamydia, the prevalence of *T. vaginalis *does not decrease with age [11-13]. Offering hope for control of this STD is the development of a new point-of-care diagnostic [11,14,15]. Given the significant human morbidity caused by *T. vaginalis*, there is an urgency towards identifying virulence factors and elucidating the mechanisms of disease pathogenesis.

Immunoglobulin A (IgA) antibodies in external secretions may be important in innate immunity and provide for specific defense against mucosal infections. IgA has been shown to be protective against viral, bacterial and parasitic infections [16-18], and inhibition of adherence and agglutination may be mechanisms for clearance [19-21]. Intestinal infection stimulates the mucosal production of secretory IgA antibodies shown to block the in vitro adherence of *Entamoeba histolytica *trophozoites to epithelial cells thereby preventing lysis of host cells and damage to tissues [22,23]. In *Giardia lamblia*, IgA antibodies act as a first line of defense for immune exclusion of the parasite at the mucosal surface [24]. Studies performed with mice have shown that protection against *Chlamydia trachomatis *genital infection is attributed to IgA. For example, there is a direct correlation between mice cervical IgA level and clearance of cervical chlamydial antigen [25]. Furthermore, IgA mAb against chlamydial major outer membrane protein confers passive protection in mice [26], and likewise, IgA mAb protects against murine experimental shigellosis [27] and oral challenge of the invasive *Salmonella typhimurium *[18].

In this report we identify a novel 44-kDa (TV44) surface protein of *T. vaginalis *that is recognized by an IgA mAb. The *tv44 *is single copy in the trichomonad genome and is yet another gene found to be down-regulated at the transcriptional and translational levels among iron-depleted organisms [28-30]. Remarkably, in contrast to adhesins that are increased in amounts and surface expression upon contact with immortalized vaginal epithelial cells (VECs) [29,31], decreased synthesis and amounts of surface TV44 follow interaction of parasites with VECs. This makes TV44 of *T. vaginalis *another member of proteins, including adhesins and α-actinin, with at least two independent signaling pathways involving iron and VEC adherence [32]. Lastly, the gene and surface protein were also detected in the bovine trichomonad responsible for fetal wastage, *T. foetus*, and the oral trichomonad, *T. tenax*.

## Results

### IgA detects a surface protein of *T. vaginalis*

**Figure 1:**
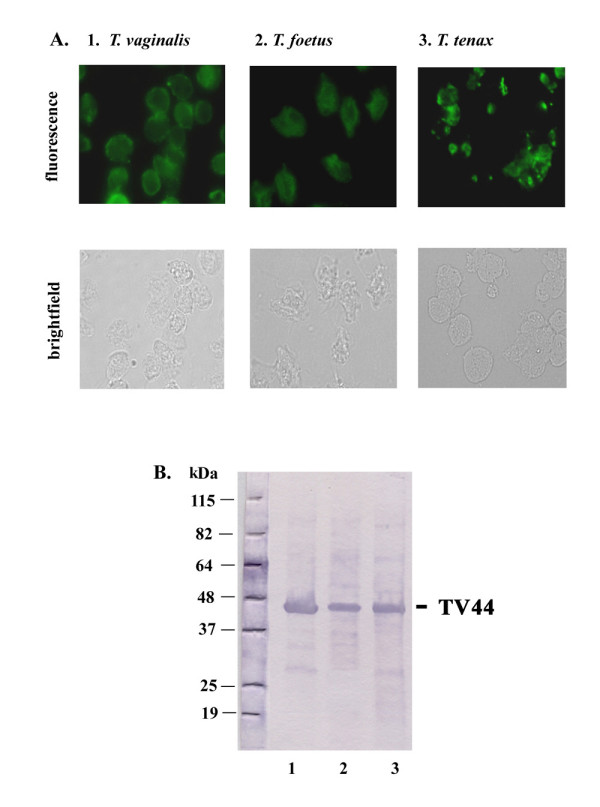
***T. vaginalis *surface immunofluorescence by monoclonal antibody (mAb) 6B8 and immunoblot detection of a trichomonad protein**. A. Fluoresceine isothiocyanate- conjugated goat anti-IgA antibody reacts with the surface of non-permeabilized *T. vaginalis *(A1), *T. foetus *(A2), and *T. tenax *(A3) incubated with IgA mAb 6B8. Brightfield microscopy is provided below the fluorescence panels to illustrate the integrity of the organisms. Fluorescence experiments were performed at least 5 times, and the distinct patchy pattern of fluorescence for *T. tenax *contrasted with the similar overall surface fluorescence for *T. vaginalis *and *T. foetus*. B. Total proteins of 10^7 ^trichomonads of *T. vaginalis *(lane 1), *T. foetus *(lane 2), and *T. tenax *(lane 3) were separated on 10% polyacrylamide gels by SDS-PAGE prior to blotting onto Hybond-P™ membranes. Blots were probed with mAb 6B8. Molecular weight of the protein is indicated in kilodaltons (kDa) (×1000).

The IgA mAb 6B8 reactive with trichomonads in a WC-ELISA was used in immunofluorescence experiments. Not surprisingly and as can be seen in Figure 1, mAb 6B8 gave a strong intensity of uniform fluorescence over the surface of *T. vaginalis *organisms (panel A1). No fluorescence was ever observed with secondary antibody alone, as before, [29,31]. Interestingly, mAb 6B8 also gave uniform surface fluorescence with *T. foetus*, the bovine trichomonad (panel A2). A different pattern of surface fluorescence was observed for *T. tenax*, the oral trichomonad (panel A3). Next, we probed nitrocellulose blots of total proteins after SDS-PAGE with mAb 6B8. Figure 1B shows a single band of identical mobility identified by the IgA mAb in *T. vaginalis *(lane 1), *T. foetus *(lane 2) and *T. tenax *(lane 3), indicating the presence of a similar-sized immuno-crossreactive protein among the human and bovine trichomonads. As controls, no proteins were detected in nitrocellulose blots of total proteins handled identically but in the absence of mAb, as before [29,31].

### Isolation of the tv44 cDNA and sequence homology comparisons of TV44

**Figure 2:**
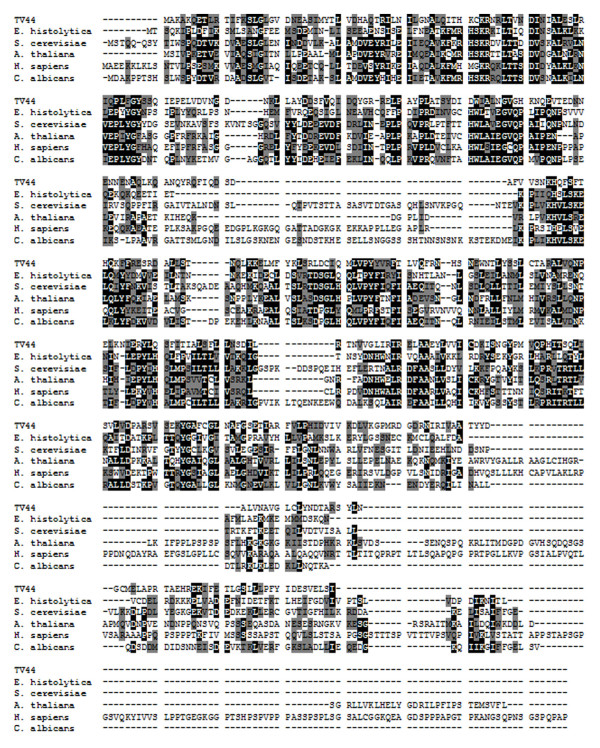
**TV44 amino acid sequence alignment with proteins of BLAST search**. The corresponding proteins of *Entamoeba histolytica *[XM 645307.1], *Saccharomyces cerevisiae *[P 53040], *Arabidopsis thaliana *[AY463621.1], *Homo sapiens *[AAH18115], and *Candida albicans *[XM 711022.1] were aligned by the Clustal W program. The conserved residues are indicated by black shading, and similarity is indicated by grey shading. TV44 had partial homology to transcription initiation factor (TFIID) of *E. histolytica *(13% identity) and *S. cerevisiae *(16%). TV44 had homology to the TATA-binding protein associated factor of *A. thaliana *(15%) and *H. sapiens *(13%) and, in addition, to the RNA pol II transcription factor of *C. albicans *(17%).

We next screened a cDNA expression library using mAb 6B8. Three of eight clones encoding recombinant proteins detected by mAb 6B8 were sequenced, and one clone with an insert size of 1422-bp represented a full-length cDNA with an open reading frame (ORF) of 1314-bp [Gene bank: AY963297] encoding a protein of 438 amino acids. The *tv44 *coding region was PCR amplified for *T. vaginalis*, *T. foetus *and *T. tenax*, and the DNA sequences had 100% identity, indicating the complete conservation of the *tv44 *gene among the human and bovine trichomonads (data not shown). Furthermore, we performed a BLAST search in the NCBI data bank for sequence homology, and Figure 2 shows the alignment of the complete sequence with 5 proteins that gave the highest percent of homology ranging from 13% to 17%. Interestingly, these proteins represented transcription initiation factors, albeit the significance of this homology for function of TV44 is unknown.

### The tv44 is a single copy gene

**Figure 3:**
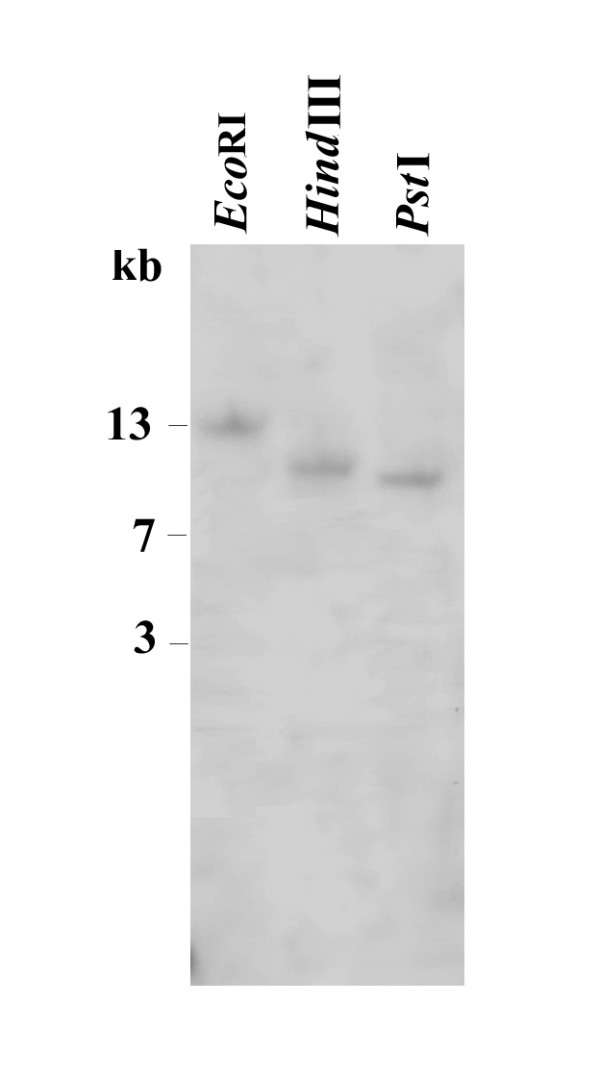
**Southern blot analysis of *T. vaginalis *genomic DNA**. Ten micrograms of genomic DNA was digested with restriction enzymes before separating on 0.9% agarose gels and blotting onto Hybond™ -N+ membranes. The membranes were then probed with a labeled PCR product containing the coding region of the *tv44 *gene. Numbers on the left show the positions of a 1-kilobase (kb) ladder.

We then performed Southern analysis of the gene in the *T. vaginalis *genome. Genomic DNA was restricted with *Eco*RI and *Hind*III, which cut outside the gene and *Pst*I that digests close to the 3'-end of the gene. Blots were probed with a PCR-derived 1.3-kb radiolabeled fragment representing the coding region. As presented in a representative blot in Figure 3, a single hybridizing band in the different restriction digests was obtained for *tv44*. No additional band was evident for the *Pst*I-digested DNA, which is likely due to hybridization of the probe with the larger full-length DNA gene fragments coupled with a diminished signal from a 3'-end fragment with only a small portion of the gene. This data suggests that *tv44 *is a single copy gene in *T. vaginalis *genome.

### Independent down-regulation of TV44 in parasites grown in low-iron medium and after contact with immortalized VECs

**Figure 4:**
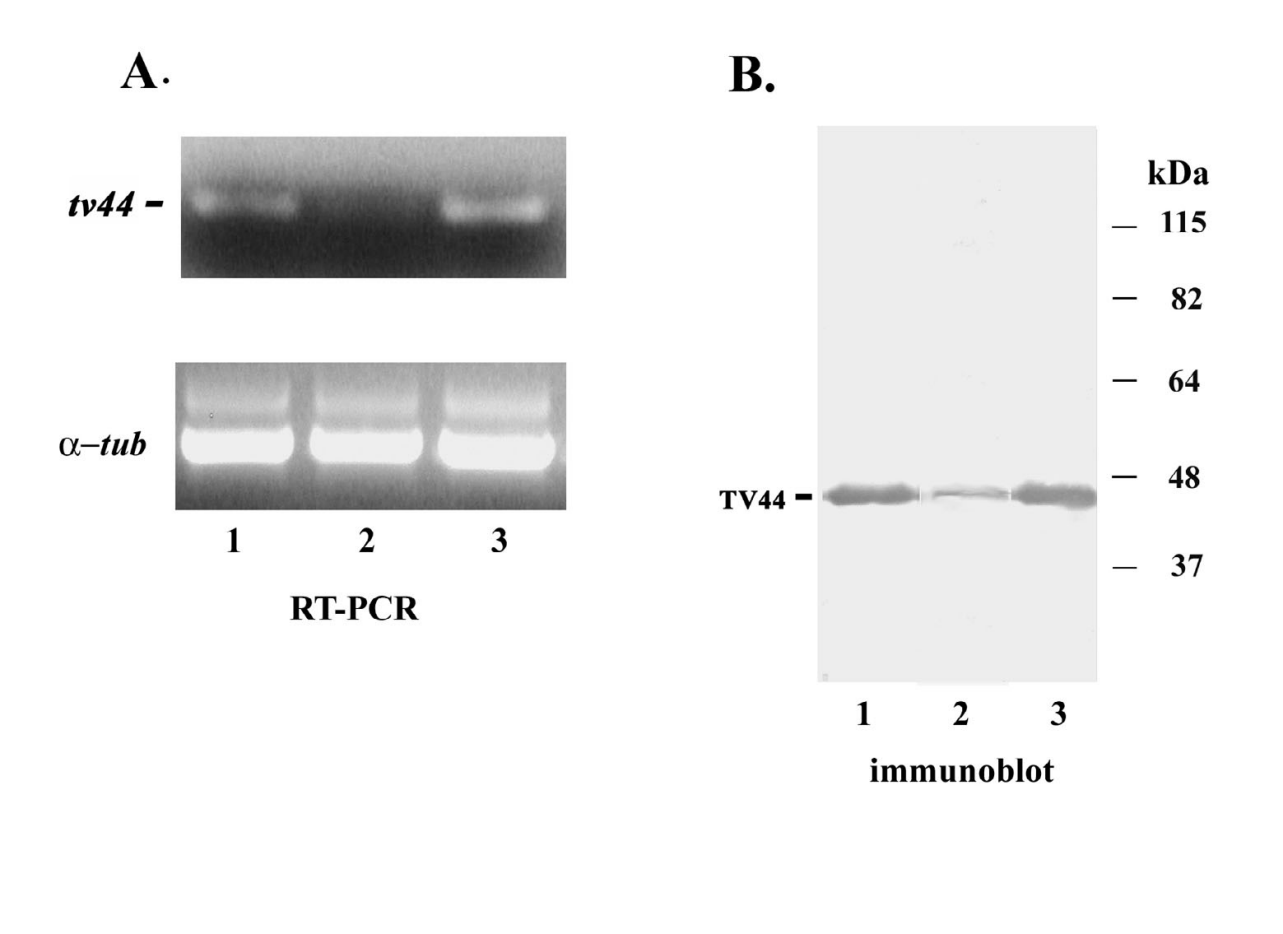
**The *tv44 *gene expression is down-regulated by low iron**. A. Ethidium bromide-stained agarose gels after electrophoresis of RT-PCR products for the *tv44 *transcript in parasites grown in normal medium (lane 1) or in low- (lane 2) versus high-iron (lane 3) medium. The bottom panel shows the RT-PCR products for the *T. vaginalis *α-tubulin (*α*-*tub*) gene as a control to show equal amounts of RNA in the PCR reactions. B. Immunoblot detection by mAb 6B8 of a 44-kDa protein after SDS-PAGE and blotting as described in the legend of Figure 1 of total proteins derived from trichomonads grown in normal medium (lane 1) and high- (lane 2) versus low-iron (lane 3) medium, as indicated. Numbers on the right are molecular weight standards in kilodaltons (× 1000).

Trichomonads were grown in medium replete with or depleted of iron and compared to organisms cultured in normal medium. RT-PCR was performed on total RNA using primers to amplify a 550-bp fragment of the *tv44 *coding region (Figure 4A). The amount of the *tv44 *transcript PCR product was decreased in parasites grown in low-iron medium (lane 2) compared to organisms cultured in normal (lane 1) or high-iron (lane 3) medium. As a control, PCR was performed using primers to α-tubulin (*α*-*tub*), and equal amounts of transcript were obtained regardless of the iron status of the parasites, showing equivalent amounts of RNA in the reactions. The amounts of TV44 detected by mAb 6B8 in nitrocellulose blots of total proteins from equal number of trichomonads corresponded to the RT-PCR data. As presented in Figure 4B, TV44 was decreased in amounts in trichomonads grown in low-iron medium (lane 2) compared to parasites grown in normal (lane 1) and high-iron (lane 3) medium, which had equivalent amounts of protein. Equal amounts of protein from identical numbers of parasites were electrophoresed and blotted. These data suggest that expression of the *tv44 *gene is regulated by low-iron medium conditions.

**Figure 5:**
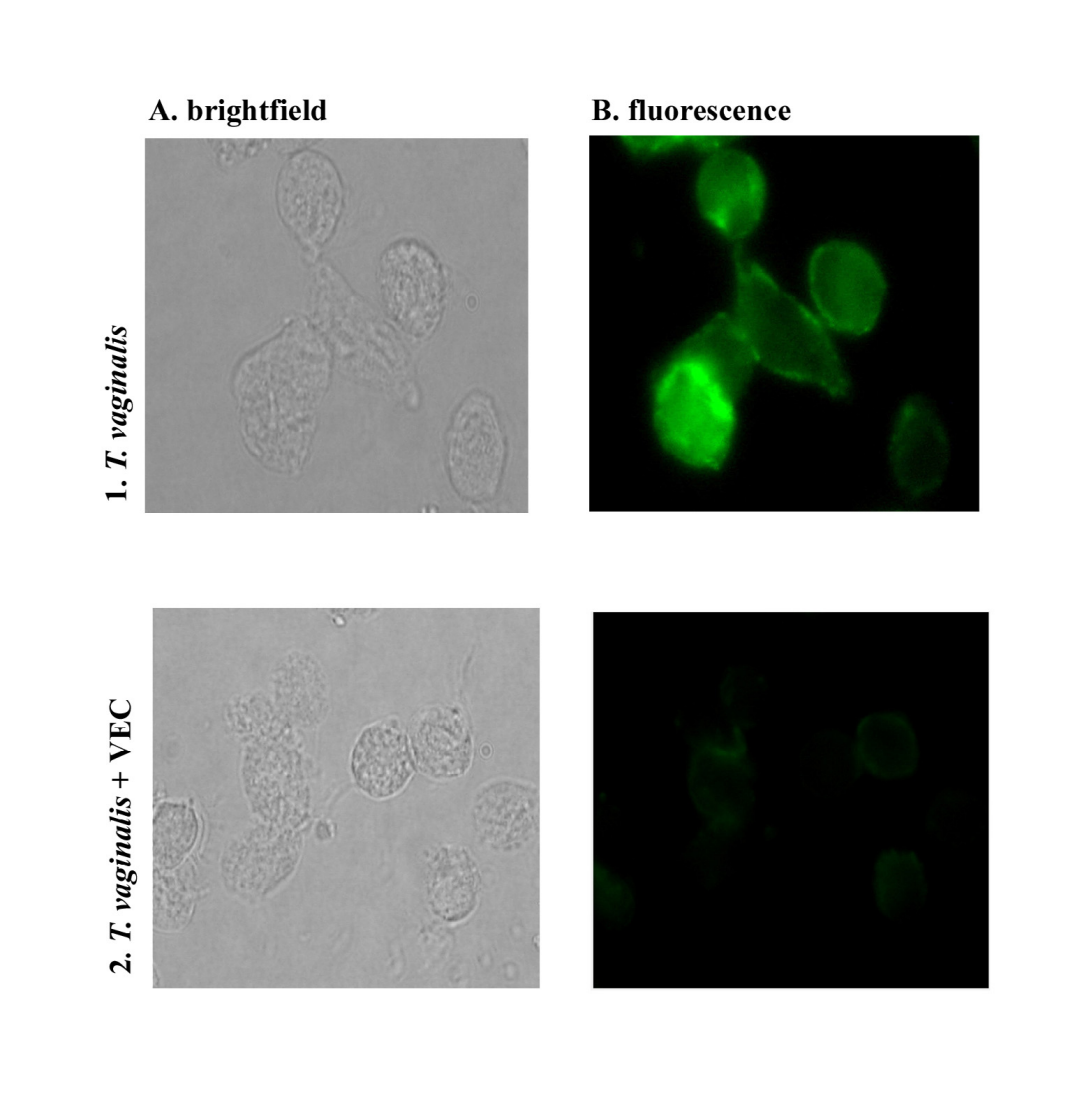
**Surface expression of TV44 on trichomonads decreases after contact with VECs**. FITC-conjugated goat anti-IgA antibody bound to IgA mAb 6B8 on the surface of non permeabilized *T. vaginalis *organisms as evident by uniform fluorescence seen in panel B1. In contrast, parasites isolated after contact with MS-74 VECs and handled identically had little or no detectable fluorescence as seen in panel B2. Brightfield pictures show the integrity of parasites used for the assay. No fluorescence was detectable in the absence of the mAb 6B8 as negative controls (not shown).

**Figure 6:**
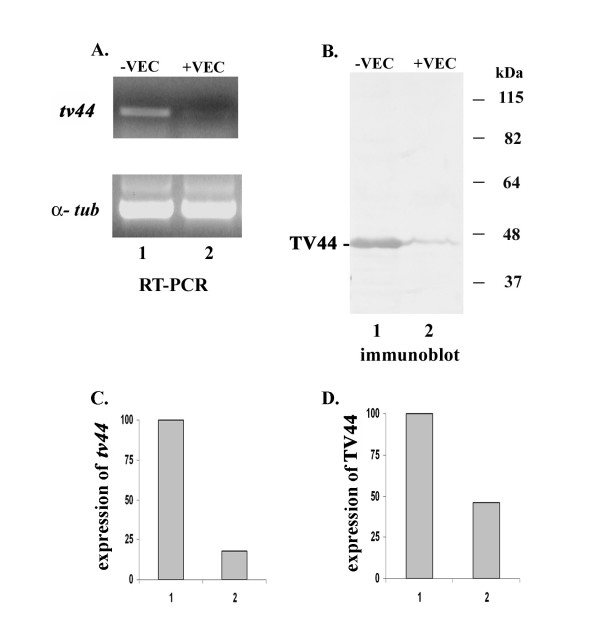
***T. vaginalis *contact with VECs results in decreased *tv44 *transcription (A and C) and amounts of TV44 (B and D)**. A. Decreased amounts of *tv44 *RT-PCR products as evidenced by ethidium bromide-stained gels after electrophoresis in 1% agarose from trichomonads after contact with immortalized MS-74 VECs (lane 2) compared to parasites without contact to VECs (lane 1). The equal quantities of the α-tubulin (*α*-*tub*) RT-PCR products show equal amounts of RNA in the PCR reactions. B. Decreased amounts of TV44 detected by mAb 6B8 on immunoblots of total proteins of trichomonads after contact with VECs (lane 2) compared to control organisms (lane 1). Proteins for SDS-PAGE were from equal numbers of organisms as described in the legend of Figure 1C and D. The relative amounts of the *tv44 *gene RT-PCR product in stained agarose gels (A) and TV44 on immunoblots (B) were obtained by scanning the bands from parts A and B using the Scion image beta program. The amounts of PCR product and protein obtained from parasites without contact with VECs was normalized to 100% (bars1) for comparison with PCR product of organisms after contact with host cells (bars 2).

We recently demonstrated the up-regulation of parasite genes following adherence of trichomonads to immortalized human VECs [32]. Therefore, we tested for the expression of TV44 following contact of normal grown trichomonads with immortalized MS-74 VECs. To our surprise as shown in Figure 5 and in contrast to adhesins and other genes increased in expression [32], we observed an immediate decreased fluorescence by mAb 6B8 after adherence (panel B2) when compared to trichomonads handled identically in the absence of VECs (panel B1). We then performed combined RT-PCR and immunoblot analysis, and *T. vaginalis *organisms after contact had decreased amounts of both *tv44 *PCR product (Figure 6A, lane 2) and TV44 protein (Figure 6B, lane 2) in contrast to duplicate trichomonads without VECs (lane 1). Amounts of RT-PCR product and protein were decreased 80% (Figure 6C, bar 2) and 53% (Figure 6D, bar 2), respectively, when compared to controls (bar 1). As a control we showed equal amounts of RNA for the RT-PCR reaction by performing RT-PCR with primers specific to α-tubulin (*α*-*tub*). Further, equivalent amounts of total proteins for identical number of parasites without and with VECs was evident by equal intensities of duplicate stained gels. As these experiments were done with organisms grown in normal medium, these data reinforce the notion that contact with VECs is an alternative signal to low iron in modulating expression of TV44.

## Discussion

To our knowledge, this is the first report describing IgA mAb reactivity to a surface protein of *T. vaginalis*. Other novel features of this protein are as follows: First, TV44 is identical to a protein in *T. tenax*, the oral trichomonad of humans, and in *T. foetus*, the bovine trichomonad responsible for fetal wastage [33,34]. Second, TV44 becomes a member of other trichomonad protein families down-regulated in expression by low-iron, such as the adhesins [28,29]. Third, expression of TV44 is down-regulated independently by either growth of organisms in a low-iron environment or by trichomonads grown in normal medium after contact with host VECs. In this respect, the *tv44 *gene and TV44 represent members of other genes and proteins, like adhesins and α-actinin, with distinct regulatory networks involving iron and adherence [32]. Therefore, it is now evident that multiple signaling pathways are functional for distinct surface proteins of *T. vaginalis*. It is likely that structure-function characterization of TV44 will help determine the significance of these two distinct regulatory pathways for this surface protein. Nonetheless, this finding makes clear that *T. vaginalis *responds during infection both to environmental cues like iron and to adherence to the vaginal epithelium by up- and down-regulating expression of genes [32], a theme that has been consistent for this mucosal pathogen.

With regard to function, the low percent homology of TV44 with higher eukaryotic transcription initiation factors (Figure 2) may not be significant, and it would be unreasonable at this time to hypothesize such a function for TV44. Nonetheless, these are the only known proteins in the NCBI data bank to which any homology was observed. Given the surface localization and the fact that TV44 is not detected in purified nuclear extracts by immunoblot analysis (data not shown), it seems unlikely that this protein functions in transcription regulation. However, the complex regulatory pathways for gene expression of TV44 are consistent with those of other known virulence factors of *T. vaginalis *[32], and therefore, a role for this protein in virulence and pathogenesis cannot be discounted. The down-regulation in transcription and surface expression upon contact with VECs may be indicating a role for TV44 for maintenance of morphologic integrity in an ellipsoid form for non-adherent, free-swimming parasites compared to the amoeboid adherent forms [31]. Alternatively, TV44 may interfere with efficient cytoadherence thereby necessitating immediate removal of the protein from the surface upon contact, essentially involving surface remodeling by *T. vaginalis*. It is not inconceivable that a similar function may exist for the opportunistic *T. tenax *and the pathogenic bovine trichomonad. Indeed, *Neisseria meningitidis *has extensive surface remodeling [35], and *Candida albicans *undergoes both up- and down-regulation of surface proteins after host cell contact [36]. Future work will be required to fully understand the function of this contact-modulating protein in the host-trichomonad interrelationship.

The biological protective role of IgA in recognition of surface protein immunogens in trichomonosis is poorly understood. The IgA immunoglobulin has been associated with resistance to a number of mucosal pathogens [16-18,21,37]. A positive correlation has been established in *E. histolytica *between the presence of IgA antibodies and the lower incidence of amoebiasis among breast-fed children and individuals from endemic areas [38,39]. There also appears to be protection through inhibiting trophozoite adherence, a blocking ability of human IgA antibodies [40]. A mucosal IgA anti-lectin antibody response was also associated with immune protection against *E. histolytica *colonization [41]. It is significant that IgA mAb has been shown to be protective for *C. trachomatis *in rodent studies [25,26], and likewise, IgA mAb protects against murine experimental shigellosis [27] and oral challenge of the invasive *S. typhimurium *[18]. Moreover, IgA-deficient mice exhibit increased susceptibility to mycobacteria infections [37]. It is noteworthy that no inhibition of adherence was evident when trichomonads pretreated with the mAb at concentrations known to give intense fluorescence (Figure 1) were added to VECs (Methods and data not shown). This apparent absence of a role for this surface protein in adherence was not surprising given that a ligand assay to identify adhesins [29-32] did not show TV44 bound to VECs (data not shown). Furthermore, that contact with VECs results in disappearance of the surface TV44 (Figure 5) suggests only a temporal role, if any, for TV44 in any adherence event. Of interest is whether this IgA mAb can be evaluated in animal protection studies [42]. Indeed, these types of experiments may provide clues as to the importance of this protein to the biology of these parasites and the conservation of identical TV44 proteins in different human species and in the bovine trichomonads. Therefore, it is important that the nature of antibody responses in patients insofar as protection by IgA through recognition of specific surface proteins of *T. vaginalis *be better characterized in the future.

## Conclusion

This paper describes the identification and characterization of a novel *T. vaginalis *IgA-reactive surface protein called TV44. Among the unique features of this single copy gene is identity to a gene in the human oral trichomonad, *T. tenax*, and in the bovine trichomonad, *T. foetus*. The *tv44 *gene is another in this parasite regulated in expression by two independent signaling mechanisms involving iron and contact with host cells. This makes TV44 a member of a growing number of *T. vaginalis *proteins with complex regulatory networks.

## Methods

### Parasite culture

*T. vaginalis *isolate T016 [29,31] and *T. foetus *isolate 02–97 were grown in trypticase-yeast extract-maltose (TYM) medium with 10% heat-inactivated donor horse serum at 37°C [43]. *T. tenax *strain Hs-4:NIH was grown in YI-S medium from ATCC (American Type Culture Collection, Manassas, VA, USA) with 10% heat-inactivated donor horse serum at 37°C. For iron replete organisms, TYM-serum was supplemented with 200 μM ferrous ammonium sulphate (Sigma Chemical Co., St. Louis, MO), and iron depleted parasites were obtained by cultivation in medium depleted of iron with 50 μM 2, 2-dipyridyl (Sigma) [28].

### Generation of mAb (6B8) to methanol fixed *T. vaginalis*

Trichomonads at the logarithmic phase of growth suspended in cold PBS were fixed overnight at 4°C with 20% methanol. After washing and resuspending in PBS, BALB/c mice were immunized by subcutaneous injection with 0.5 ml containing 5 × 10^6 ^fixed *T. vaginalis *emulsified in Freund's complete adjuvant followed by two additional boosts of the same amount of antigen mixed with Freud's incomplete adjuvant performed on days 14 and 28. A final boost of the same amount of antigen in saline was given intravenously at day 42. The mice with higher antibody titers were used for hybridoma production using published protocols [44,45]. A whole cell-ELISA was used to identify antibodies reactive with the surface of trichomonads [44]. Hybridoma cells were single cell cloned and supernatant used for fluorescence and immunoblot assays.

### Parasite adherence

To demonstrate that contact with host cells resulted in loss of surface expression of TV44, immortalized MS-74 human VECs [46] were used for adherence experiments and were grown in Dulbecco's modified Eagle medium (D-MEM) (Invitrogen-Life Technologies, Carlsbad, CA) supplemented with 10% fetal bovine serum, at 37°C in the presence of 5% CO_2_. The immortalized human MS-74 VECs were used for adherence as recently detailed [47].

Experiments were also carried out to determine if the IgA mAb pretreatment of organisms inhibited cytoadherence. For the adherence assay, confluent monolayers of MS-74 VECs on individual 96-well microtiter plates were stabilized with 3% glutaraldehyde, as before [30], prior to addition of labeled parasites. Organisms (5 × 10^6^) at late logarithmic phase of growth were washed in PBS and suspended in 1 ml TYM without serum for labeling with calcein for 30 min at 37°C. After washing in PBS, trichomonads were suspended in 1 ml TYM medium without serum containing hybridoma supernatant with mAb 6B8 diluted 1:100. Then, 2.5 × 10^5 ^parasites washed once in PBS were added to individual wells of confluent fixed VECs and the parasite-VEC interaction incubated at 37°C [29-32]. After 30 min, the wells were washed three times with warm PBS, and adherent organisms were lysed with 200 μl of 0.1% Triton X-100. The level of adherence to VECs was determined by the intensity of fluorescence of the lysate at 494-nm with a VersaFluor Fluorometer (BioRad Laboratories, Hercules, CA).

### Immunoblot detection

Total proteins from 10^7 ^*T. vaginalis *parasites were obtained as before using trichloroacetic acid (TCA) [48] for analyses by sodium dodecylsulphate-polyacrylamide gel (SDS-PAGE) electrophoresis [49] using 10% acrylamide prior to blotting onto Hybond-P™ membranes (Amersham Pharmacia Biotech, Piscataway, NJ). Blots were blocked in 0.1% Tween 20 and 5% BSA before probing with mAb 6B8. The blots were further incubated with goat anti-mouse IgA secondary antibody conjugated with alkaline phosphatase (Sigma). Then, the blots were washed well and incubated in alkaline phosphatase substrate (Sigma) to visualize the reactive band.

### Isolation of *T. vaginalis *nuclear extract and immunoblot analysis

For purification of *T. vaginalis *nuclear extracts by an established protocol [50], organisms were pelleted by centrifugation at 1,500 × g for 10 min at 4°C. Trichomonads were washed twice with PBS and suspended in 5 cell volumes of buffer (20 mM HEPES-KOH, pH 7.6, 10 mM KCl, 1.5 mM MgCl_2_, 0.1 mM EDTA, 1 mM DTT, 25 mM leupeptin, 52 mM N-α-p-tosyl-_L_-lysine chloromethyl ketone (TLCK), 1 mM phenylmethylsulphonyl fluoride (PMSF), and 0.25% NP-40. The suspension was transferred to a glass homogenizer and the organisms lysed with 10 strokes. The lysate was centrifuged at 3,000 × g for 10 min at 4°C. The nuclear pellet was suspended in 4 volumes of buffer (20 mM HEPES-KOH, pH 7.6, 420 mM KCl, 1.5 mM MgCl_2_, 0.1 mM EDTA, 1 mM DTT, 10 μg of leupeptin per ml, 50 μg of TLCK per ml, 1 mM PMSF, and 20 % glycerol) and incubated at 4°C while stirring for 30 min. The extract was then centrifuged at 100,000 × g for 1 h at 4°C and supernatant dialyzed against 1 liter of buffer (20 mM HEPES-KOH, pH 7.6, 100 mM KCl, 1.5 mM MgCl_2_, 0.1 mM EDTA, 1 mM DTT, and 20 % glycerol) o/n at 4°C. Two micrograms of nuclear extract was subjected to SDS-PAGE using 10 % acrylamide gels. After electrophoresis, proteins on gels were blotted onto Hybond-P™ membrane (Amersham Pharmacia Biotech). Immunoblot analysis was carried out with the IgA mAb 6B8, and protein bands were visualized with alkaline phosphatase-conjugated goat anti IgA antibody and substrate, using standard procedures as above.

### Immunofluorescence detection of TV44 on the parasite surface

Immunofluorescence of TV44 on the surface of non-permeabilized trichomonads was carried out using a modification of a recent procedure [29]. Briefly, 1 × 10^6 ^*T. vaginalis*, *T. foetus *and *T. tenax *parasites at the logarithmic phase of growth were washed twice with cold PBS and fixed with 4% paraformaldehyde for 30 min at room temperature (RT). Trichomonads washed in PBS were suspended in 1 ml PBS and treated with 5% BSA for 1 h at RT. Organisms were washed and resuspended again in 1 ml PBS prior to incubation for 1 h with hybridoma supernatants of mAb 6B8 diluted 1:100. Parasites were washed with PBS and incubated for 1 h at 37°C with affinity-purified goat anti-mouse IgA (α-chain specific) antibody conjugated to fluoresceine isothiocyanate (Sigma) diluted 1:100. Finally parasites were washed twice with PBS and observed under 100× magnification using the Olympus BX41 microscope.

### cDNA library construction

*T. vaginalis *isolate T016 [29,31] cDNA library was constructed in the Lambda Zap II vector from 5 μg poly (A)+ RNA using the poly(A) Quick mRNA isolation kit and the cDNA synthesis kit (both from Stratagene, La Jolla, CA). Immunological screening with the mAb 6B8 was carried out as described [51]. After two rounds of screening and plaque purification, phagemids were excised with Exassist interference-resistant helper phage according to the manufacturer's instructions. Sequencing was performed at the Advanced Nucleic Acid Core Facility of University of Texas Health Science at San Antonio.

### Computer analysis of sequence data

The nucleotide sequence of the cDNA clone was translated into the corresponding amino acid sequence with BioEdit program. The BLAST program was used to find related proteins in the data bank [52]. Sequences were aligned using Clustal W [53] program.

### RNA isolation and RT-PCR

Total RNA was isolated using the trizol reagent (Invitrogen-Life Technologies). In vivo expression of *tv44 *gene was analyzed by semi-quantitative RT-PCR. Total RNA from iron depleted and iron enriched *T. vaginalis *parasites was reverse transcribed with oligo (dT)_15 _primer using Superscript II reverse transcriptase (Invitrogen-Life Technologies). PCR amplification of cDNA was carried out using the following *tv44 *specific primers: sense primer (5'- GGTCTGATCCGCATCAGAGAG-3') and antisense primer (5'-CTCTCTGTGTT CAGCTGTTC-3'). Trichomonad α-tubulin gene was used as an internal control. The PCR was performed for 25 cycles.

### Genomic DNA isolation and Southern blot

Genomic DNA from *T. vaginalis *was obtained as described [54]. For Southern blot hybridization analysis, genomic DNA (10 μg) was digested with restriction endonucleases *Eco*RI and *Hind*III that digests outside the gene and *Pst*I that cuts once inside the gene ~200 nucleotides of the 3'-end stop codon. Digested DNA was electrophoresed on 0.9% agarose gel. DNA was transferred on to Hybond™-N+ membrane (Amersham Pharmacia Biotech) using a vacuum blotter (Boekel Scientific, Feasterville, PA). Hybridization was performed by following the standard procedure [51]. The probe represented the entire coding region of TV44 and was generated by PCR and labeled with ^32^P using Quick prime labeling kit (Amersham Pharmacia Biotech).

### PCR amplification of tv44 from *T. foetus *and *T. tenax*

To isolate the coding regions of TV44 from *T. foetus *and *T. tenax*, a common sense (5'- TATGACGTCGACGGGATGGCGAAAGCGAAG-3') and antisense (5'-CTATC TAAGCTTATA GTAAATCGACAATTC-3') primers were designed based on the *tv44 *sequence [Gene Bank: AY 963297]. Five hundred nanograms of *T. vaginalis *genomic DNA was used per 50 μl standard PCR reactions. The PCR products were separated on an agarose gel, and the DNA was purified using the QIA quick Gel Extraction Kit (Qiagen, Inc., Valencia, CA). After DNA sequencing, the sequences were compared to the *T. vaginalis tv44 *gene sequence.

### Reproducibility of experiments

Unless otherwise stated in the text, all experiments were performed numerous times and no less than on three different occasions.

## List of abbreviations

IgA, immunoglobulin A; D-MEM, Dulbecco's modified Eagle medium; kb, kilobase; kDa, kilodalton; SDS-PAGE, sodium dodecylsulfate-polyacrylamide gel electrophoresis; TYM, trypticase-yeast extract-maltose; MS-74 VECs, immortalized vaginal epithelial cells.

## Authors' contributions

VM designed the study and carried out the cDNA library construction, library screening, Southern and immunoblot analyses, and RT-PCR. ASK performed immunofluorescence assays and generated *T. vaginalis *parasites with and without contact with VECs. THC generated the hybridoma producing the mAb 6B8. JFA participated in the design of the experiments, offered suggestions during the experiments, and helped VM to write the manuscript. All authors read and approved the final manuscript.
